# Advancing deep learning-based segmentation for multiple lung cancer lesions in real-world multicenter CT scans

**DOI:** 10.1186/s41747-025-00617-7

**Published:** 2025-08-18

**Authors:** Xavier Rafael-Palou, Ana Jimenez-Pastor, Luis Martí-Bonmatí, Carlos F. Muñoz-Nuñez, Mario Laudazi, Ángel Alberich-Bayarri

**Affiliations:** 1 Research and Frontiers AI, Quibim, Valencia, Spain; 2 Radiology Department, La Fe University and Polytechnic Hospital, Valencia, Spain; 3 Biomedical Imaging Research Group (GIBI230) La Fe Health Research Institute, Valencia, Spain; 4 Diagnostic Imaging Unit, Department of Biomedicine and Prevention, University of Rome Tor Vergata, Rome, Italy

**Keywords:** Artificial intelligence, Deep learning, Lung neoplasms, Neural networks (computer), Tomography (x-ray computed)

## Abstract

**Background:**

Accurate segmentation of lung cancer lesions in computed tomography (CT) is essential for precise diagnosis, personalized therapy planning, and treatment response assessment. While automatic segmentation of the primary lung lesion has been widely studied, the ability to segment multiple lesions per patient remains underexplored. In this study, we address this gap by introducing a novel, automated approach for multi-instance segmentation of lung cancer lesions, leveraging a heterogeneous cohort with real-world multicenter data.

**Materials and methods:**

We analyzed 1,081 retrospectively collected CT scans with 5,322 annotated lesions (4.92 ± 13.05 lesions per scan). The cohort was stratified into training (*n* = 868) and testing (*n* = 213) subsets. We developed an automated three-step pipeline, including thoracic bounding box extraction, multi-instance lesion segmentation, and false positive reduction via a novel multiscale cascade classifier to filter spurious and non-lesion candidates.

**Results:**

On the independent test set, our method achieved a Dice similarity coefficient of 76% for segmentation and a lesion detection sensitivity of 85%. When evaluated on an external dataset of 188 real-world cases, it achieved a Dice similarity coefficient of 73%, and a lesion detection sensitivity of 85%.

**Conclusion:**

Our approach accurately detected and segmented multiple lung cancer lesions per patient on CT scans, demonstrating robustness across an independent test set and an external real-world dataset.

**Relevance statement:**

AI-driven segmentation comprehensively captures lesion burden, enhancing lung cancer assessment and disease monitoring

**Key Points:**

- Automatic multi-instance lung cancer lesion segmentation is underexplored yet crucial for disease assessment.
- Developed a deep learning-based segmentation pipeline trained on multi-center real-world data, which reached 85% sensitivity at external validation.
- Thoracic bounding box and false positive reduction techniques improved the pipeline’s segmentation performance.

**Graphical Abstract:**

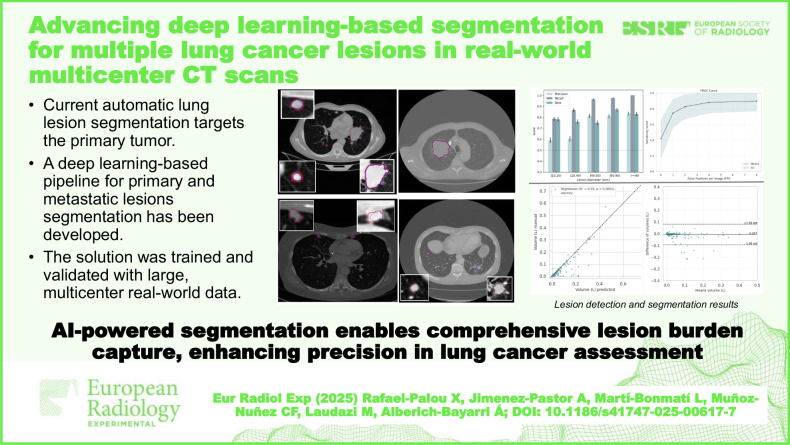

## Background

Lung cancer, particularly non-small cell lung cancer (NSCLC) is the most aggressive and prevalent form, accounting for over 80% of cases and a 5-year survival rate of 28% [1]. Computed tomography (CT) is the standard of care for staging and monitoring NSCLC, enabling physicians to identify and manually delineate the anatomical structure of the cancerous lung lesions [2]. The RECIST 1.1 guidelines standardize clinical assessments, defining measurable lesions as those with a minimum diameter of 10 mm on CT scans [3]. However, the reliance on manual and subjective measurements limits efficiency and reproducibility [4, 5].

Robust, automated lung lesion segmentation tools integrated into the radiological workflow can enhance diagnostic consistency, reduce interobserver variability, and alleviate clinical workload [6]. Yet, building fully automatic solutions remains challenging, due to limited annotated data, image acquisition variability, and the span of lesion characteristics. To tackle these issues, numerous studies [7] have leveraged in-house and publicly available lung cancer cohorts [8–10], employing ad-hoc deep learning solutions–mainly U-Net convolutional networks [11–14], with a growing interest in transformer architectures [15, 16]. Alternatively, the emergence of open and available segmentation frameworks (like nn-UNet [17]), has also contributed to effective solutions [18].

Despite these advances, a key challenge remains in translating such methods into reliable clinical deployments [19], as they are predominantly developed on controlled study populations with limited case diversity, few clinical centers, and focused on segmenting the primary lung cancer lesion [8–10]. These constraints overlook real-world imaging variability, limiting model generalizability and clinical utility. Particularly, restricting segmentation to primary lesions rather than all cancerous lesions in the lungs, prevents a comprehensive assessment of total lesion count, spatial distribution, and disease burden, which is crucial for staging, prognosis, disease progression, and treatment planning [10, 20].

This study addresses the challenge of automatic multi-instance lung lesion segmentation using a heterogeneous, multicenter lung cancer cohort. The dataset encompasses standard-of-care clinical studies and real-world CT exams from diverse clinical scenarios, such as lung cancer screening, follow-up image examinations, and whole-body CT scans. This comprehensive collection assembles a wide range of imaging manufacturers and acquisition protocols, including varying pixel spacings, and diverse field of views (FoVs). While this heterogeneity is essential for assessing the robustness and clinical applicability of the method, it also increases lesion detection complexity and the risk of false positives (FPs) [21]. To overcome these limitations, we develop and validate a novel deep learning-based pipeline specifically designed for multi-instance lung cancer lesion segmentation in a diverse cohort. We hypothesize that achieving accurate segmentations with our approach will provide a robust tool for supporting clinicians in their clinical routine.

## Materials and methods

**Fig. 1:**
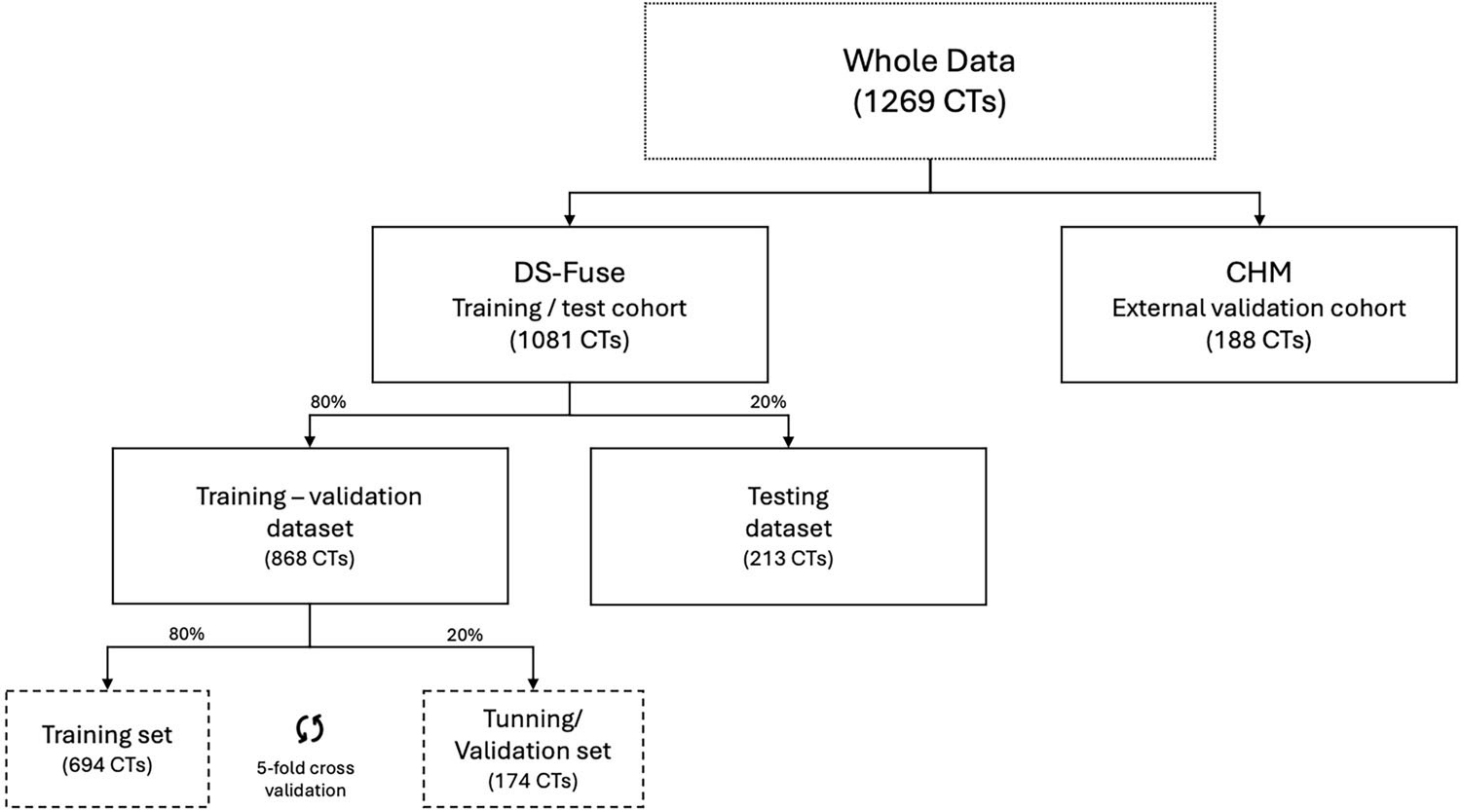
Datasets for training, validation, and testing of the proposed pipeline

This multicenter retrospective study was approved by the appropriate institutional ethics committees and conducted in compliance with the Declaration of Helsinki. Given the retrospective design, the requirement for informed consent was waived. Figure 1 provides an overview of the data distribution.

### Model development dataset

In total, 1,081 CT scans corresponding to unique subjects from three distinct data sources were collected in a cohort (DS-Fuse) for training and testing the proposed method. The first dataset (DS_0), consisting of pretreatment real-world scans acquired from 4 different manufacturers, included 98 contrast-enhanced cases, with patient ages ranging from 37 to 87 and a female ratio of 34%, collected between 2020 November and 2023 February. The second (DS_1) and third (DS_2) datasets consisted of post-treatment follow-up CT examinations, drawn from two different clinical studies. DS_1 included 236 cases, of which 99 were acquired without contrast agent (CA), from 78 clinical sites, 5 manufacturers, and 59 scanner models. Data was collected from October 2018 to July 2020, the age of the subjects was between 36 and 90 years, and the female ratio was 29%. DS_2 included 747 cases, 39 of which were acquired without CA, from 173 clinical sites and 8 manufacturers, collected from 2015 April to 2017 February. The age of the subjects was between 30 and 89 years, and the female ratio was 32%.

Visible solid lung lesions, encompassing both primary tumors and metastases, were manually delineated from the entire cohort by a team of three certified medical imaging specialists, each holding an advanced technical degree in Diagnostic Imaging and Nuclear Medicine. All team members had over six years of professional experience in clinical imaging interpretation and annotation and were supervised by a senior thoracic radiologist with over 15 years of experience. The reference standard for lesion delineation was based on established radiological criteria consistent with RECIST 1.1 [3]. Lesions were considered eligible for delineation if they were visually discernible on thin-slice CT, showed characteristics consistent with neoplastic morphology, and were considered clinically relevant based on location and size. Ambiguous cases (such as those involving atelectasis or collapse) were reviewed in close collaboration with the senior thoracic radiologist. All annotations were performed on a slice-by-slice basis using the ITK-SNAP tool [22].

The cohort was randomly stratified into training and testing datasets according to the DS (*i.e*., DS_0, 1, and 2) and spatial z-resolution reconstruction, to mitigate bias. In total, 868 cases were allocated for training (80%) and 213 cases for testing (20%). No statistically significant differences were found between training and test datasets in terms of the number of lesions per CT and lesion diameters. Table 1 provides extended details about image acquisition and lesion characteristics of the training, and test partitions.

**Table 1:** Summary of the DS-Fuse training and testing datasets

Cohort	All	Training			Test		
		DS0	DS1	DS2	DS0	DS1	DS2
CT scans							
Total	1,081	79	190	599	19	46	148
Lesion-free	81	0	0	70	0	0	11
Lung lesions (any size)							
Number of lesions	5,322	727	804	2,843	117	93	738
Number of lesions per CT	2	3	2	1	2	1	1.5
	(1–3)	(2–7)	(1–3)	(1–3)	(1.5–6.5)	(1–2)	(1–4)
Lesion diameter (mm)	14	9	13	15	8	27	14
	(10–26)	(7–15)	(9–24)	(10–30)	(6–23)	(14–63)	(10–25)
Lung lesions (≥ 10 mm)							
Number of lesions	4,055	316	600	2,414	47	77	601
Number of lesions per CT	2	2	2	1	2	1	1
	(1–3)	(1–4)	(1–3)	(1–3)	(1–3)	(1–2)	(1–4)
Lesion diameter (mm)	16	16	15	16	31	36	15
	(12–38)	(12–38)	(12–41)	(12–36)	(14.5–56)	(18–75)	(12–33)
Image size (mm)							
*x*	397	377	426	398	364	384	394
	(360–437)	(350–430)	(372–438)	(360–440)	(361–440)	(339–425)	(353–410)
*y*	399	377	426	399	364	384	394
	(360–438)	(350–430)	(372–438)	(360–440)	(361–440)	(339–425)	(353–410)
*z*	470	372.5	577.2	485	314.5	445	646
	(364.5–646)	(324–615)	(370–627)	(385–651)	(280–614)	(315–642.5)	(385–673.75)
Voxel size (mm)							
*x*	0.77	0.73	0.82	0.77	0.71	0.75	0.76
	(0.70–0.85)	(0.68–0.83)	(0.72–0.85)	(0.70–0.85)	(0.70–0.85)	(0.66–0.83)	(0.68–0.80)
*y*	0.77	0.73	0.82	0.77	0.71	0.75	0.76
	(0.70–0.85)	(0.68–0.83)	(0.72–0.85)	(0.70–0.85)	(0.70–0.85)	(0.66–0.83)	(0.68–0.80)
*z*	2.5	1.5	3.6	3.0	0.5	4.0	2.0
	(1.2–5)	(0.5–1.5)	(2–5)	(1.2–5)	(0.5–1.5)	(3–5)	(1.2–5)

Continuous variables are expressed as medians (interquartile ranges)

### External validation dataset

For external validation, a pretreatment real-world cohort of 188 contrast-enhanced CT scans (DS-CHM) was retrieved from the ChAImeleon European project [23], with approval from the corresponding ethical committee. The data was collected between January 2015 and January 2022; the age of the subjects was between 35 years to 83 years, with a female ratio of 60%. CT scans were acquired from 4 different manufacturers, with a median slice thickness of 2.0 mm (interquartile range [IQR] 1.0–3.0 mm) and FoV of 415 mm (IQR 379–441 mm). Further cohort details in Supplementary Material, Section S1.

### Segmentation pipeline

The proposed end-to-end pipeline (LLSB_CFPR) was composed of three steps: thoracic bounding box, multi-instance lesion segmentation network, and FP reduction.

#### Thoracic bounding box

**Fig. 2:**
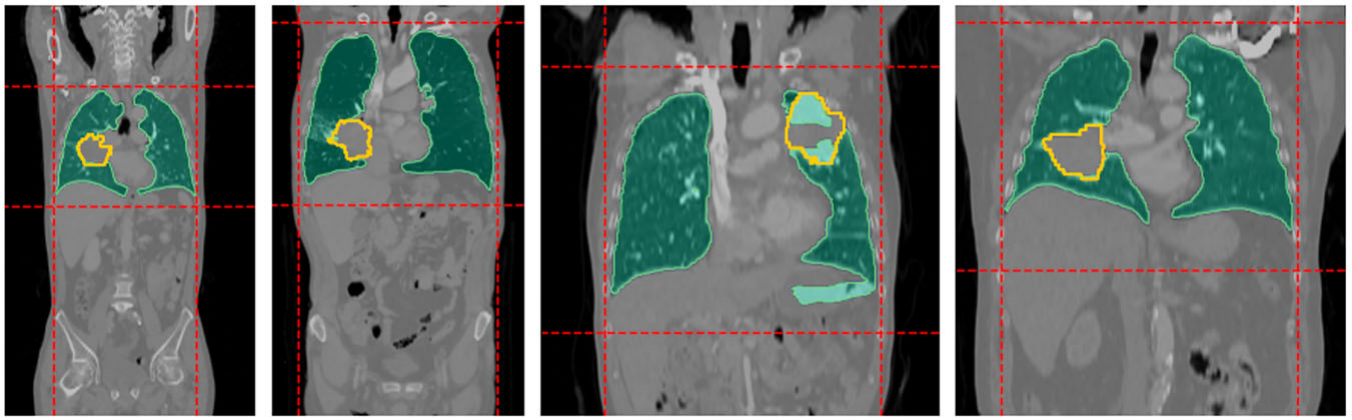
Examples from the cohort (with different fields of view) where the automatically generated lung masks (green) failed to encompass ground truth lesions (orange), while the predicted thoracic bounding boxes (red dashed lines) successfully captured them

A preprocessing step was implemented to isolate the thoracic area by cropping the input images based on automatically generated masks from the lungs. This ensured the analysis remains focused on the anatomical area of interest, reduced computational cost from large FoVs included in our cohort, and preserved compatibility with CT scans of arbitrary FoVs. To this end, we used LMInferer [24], an open-source tool based on a U-Net architecture [11] for lung segmentation. However, directly using outputs from this tool was proved suboptimal in cases with large masses or lesions attached to the walls (see Fig. 2). To sort out this limitation, a three-dimensional (3D) bounding box surrounding the predicted lung masks was computed. Specifically, we binarized the predicted masks and then determined the corner coordinates of the segmented region along each spatial dimension. To mitigate the impact of lung segmentation errors on subsequent pipeline steps, a set of minimum values for each dimension of the bounding box (*x*, *y*, and *z)* was empirically determined from the smallest lungs in the training set (*i.e*., 21 cm, 15 cm, and 12 cm). Therefore, if any dimension of the predicted bounding box in an unseen case was less than the minimum value for that dimension, the dimension of the whole image was used instead.

#### Multi-instance lesion segmentation network

Building on prior approaches [18], we employed no new U-Net (nnU-Net), a state-of-the-art medical segmentation framework, to develop a lung lesion segmentation model [17]. However, the default nnU-Net configuration required tailored modifications to accommodate our specific multi-instance segmentation task. We utilized the 3D full-resolution architecture to preserve contextual and volumetric information while minimizing inter-slice inconsistencies. The network was adapted to generate binary masks, where 0 represents normal tissue and 1 corresponds to lung lesions. Additionally, we replaced nnU-Net’s default post-processing step with a conventional 3D connected-components algorithm (26-connectivity) [25] to accurately label and differentiate individual lesions. The CT scan cropped to the thoracic area was used as input to the nnU-Net. Finally, padding was added to the predicted mask to ensure the same size as the original CT scan.

#### FP reduction

**Fig. 3:**
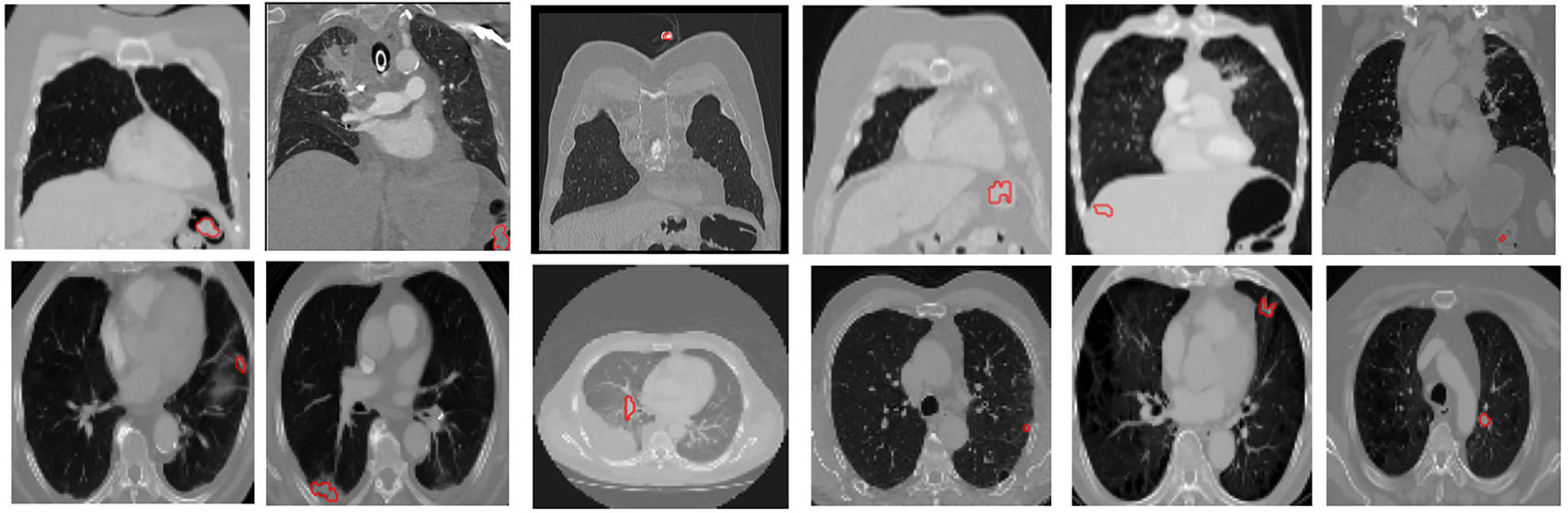
Examples of false-positive predicted segmentations (red). The top row shows predictions in the extrapulmonary region, while the bottom row shows segmentations entirely or partially within the intrapulmonary region

To enhance segmentation reliability, a postprocessing strategy was implemented to reduce FP detections from the segmentation stage. These artifacts typically arise entirely (or partially) in the intrapulmonary region, where normal lung structures can resemble cancerous lesions [21]. However, FPs were also observed in extrapulmonary areas (Fig. 3).

**Fig. 4:**
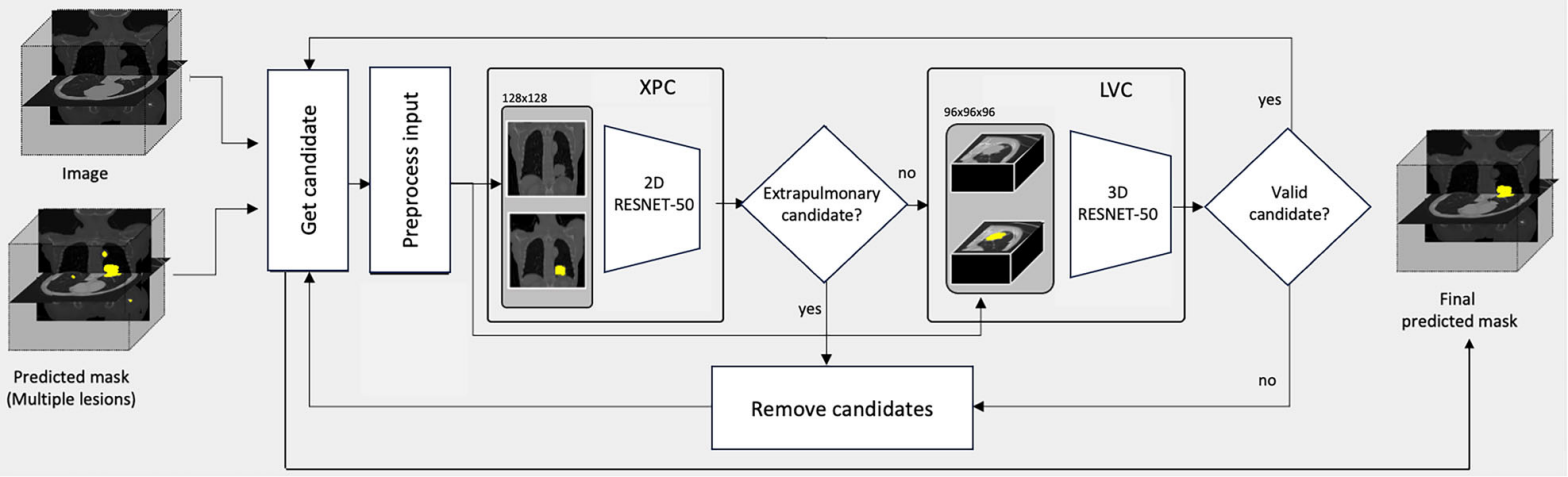
Flow diagram of the CFPR method. The XPC and LV classifiers receive tailored (2D and 3D) input patches of the same lesion candidate. The predicted masks have the raw image overlayed for visibility purposes only

To address this problem, a cascade false-positive reduction (CFPR) method was proposed, incorporating two distinct classifiers: XPC and LVC (Fig. 4). The XPC classifier filters fully extrapulmonary detections, while the LVC distinguishes valid lung lesions from FPs, assuming all remaining candidates are at least partially intrapulmonary. The process begins with a CT image and its predicted mask. Each segmented lesion candidate is extracted, and two pairs of patches are generated to meet the classifiers’ input. The XPC classifier first assesses whether a candidate is fully extrapulmonary; if its probability is below a threshold (*thr_e)*, the lesion is further analyzed by the LVC classifier. If the LVC classifier’s probability surpasses a second threshold (*thr_i*), the candidate is confirmed as a lung lesion; otherwise, it is classified as an FP and removed from the mask. This process is repeated for each predicted candidate.

The main advantage of this two-step approach lies in optimizing each classifier for its specific subtask. Following prior studies [21], both classifiers were trained using raw and predicted mask images as input. The XPC classifier specifically utilized the central coronal slice of each lesion candidate, based on the hypothesis that this plane provides sufficient anatomical detail of pulmonary boundaries to identify extrapulmonary candidates. Conversely, various input configurations were explored for the LVC classifier to determine the most effective approach, including two-dimensional (2D) and 2.5D full-slice raw and masks images from multiple planes, as well as 3D cropped patches centered on the predicted lesion. Additionally, for both classifiers, distinct network architectures (*e.g*., ResNet [26] and Swin transformer [27]) and the use of pre-trained weights (*i.e.*, ImageNet [28], MedicalNet [29]) were investigated. A summary of the experiments conducted to identify the best XPC and LVC classifiers is provided in Supplementary Material, Section S2.

### Training process

We implemented a 5-fold cross-validation (CV), ensuring stratification by DS and spatial *z*-resolution reconstruction to develop the pipeline’s models.

### Pipeline

To construct the overall pipeline (LLSB_CFPR), all images in each CV fold of DS-Fuse underwent the thoracic bounding box extraction step to ensure proper lung cropping. This step required no image preprocessing or model training; inference was directly applied with the LMInferer tool [24] to generate the lungs mask. Using the cropped images, a segmentation model based on the nnU-Net framework [17] was trained, incorporating the adaptations for multi-instance segmentation. This framework automatically prepared the data, configured non-fixed training parameters, and optimized the model without user intervention. For comparison purposes, additional segmentation models were evaluated, using state-of-the-art architectures (UResNet [30], UNet++ [31], and Swin UNet [32]). To ensure a fair comparison of these networks with our method, the same CV and configurations that closely matched those of nnU-Net were used. Further setup details are provided in Supplementary Material, Section S2.

For ablation studies, multiple lung lesion segmentation pipelines (LLS) were designed. The LLS pipeline consisted solely of the lesions’ segmentation step, serving as a baseline. LLSB introduced lung bounding box constraints to focus the segmentation within the thoracic area. Building upon this, LLSB_XPC incorporated the extrapulmonary classifier (XPC), and LLSB_LVC integrated the lesion validator classifier (LVC). Finally, LLSB_CFPR included both the extrapulmonary and LVCs.

### FP reduction

The FP-reduction method, *i.e*., the CFPR, was developed using a dedicated dataset (DS-FP), constructed from the segmentation predictions generated by running the LLSB pipeline on the DS-Fuse dataset. The resulting DS-FP cohort comprised 9,706 lesion candidates (7,727 for training and 1,979 for testing), with 5,519 nonlesions (nonmatching with ground truth lesions) labeled as 0, and 4,187 lesions (matching with ground truth lesions) labeled as 1. This dataset was used for building the LVC classifier. For the XPC classifier, a subset from DS-FP was created (DS-FPX), comprised of 4,159 manually selected lesion candidates (3,536 for training and 623 for testing). Among these, 1,784 candidates (labeled as 0) were non-lesions, located in various extrapulmonary regions. The remaining 2,375 candidates (labeled as 1) corresponded to matching true lesions, located either entirely or partially within the lungs. Both datasets were partitioned according to the 5-fold case partitioning done in the DS-Fuse.

The preprocessing for these classifiers involved resizing input images to 128 × 128 pixels for the XPC and 96 × 96 × 96 voxels for the LVC classifiers. All images were resampled to an isotropic resolution of 1 × 1 mm for the 2D and 1 × 1 × 1 mm for 3D classifiers, with intensity values normalized to a 0–1 range. The classifiers were trained using a binary cross-entropy loss function, with random data transformations (*i.e*., translation, flip, rotation, zoom, and Gaussian noise), a learning rate of 1 × 10^-3^, weight decay of 1 × 10^-^^4^, a batch size of 32, Adam as the optimizer, and early stopping after 30 epochs without loss improvement.

### Evaluation

To evaluate the performance of the models, several metrics were computed across the 5-fold CV. For lesion detection, we used F1-score, precision, and recall, defining a true positive as a predicted lesion overlapping with a ground truth annotation (Dice similarity coefficient [DSC] > 0). An FP occurred when a predicted mask had no corresponding ground truth (DSC = 0), while a false negative occurred when a ground truth mask had no corresponding prediction (DSC = 0). For lesion segmentation, we assessed performance using the DSC at both image and lesion levels. At the image level, DSCs were averaged across all CT scans. At the lesion level, we also computed the 95th-percentile Hausdorff distance (Hd-95). Each ground truth lesion was matched with all predicted lesions, selecting the highest DSC and the lowest Hd-95 score among the matches. The final lesion-level metrics were obtained by averaging per-lesion scores.

To evaluate FP reduction, the area under the receiver operating characteristic curve (AUROC), precision, recall, and specificity were calculated between probability predictions and binary ground truths. For volume quantification agreement, the coefficient of determination (*R*^*2*^), and the Bland–Altman analysis were used to obtain the bias to quantify the systematic error, and the limits of agreement between the measures.

To estimate the uncertainty of the performance metrics, we employed bootstrapping with 1,000 resamples, reporting the mean and 95% confidence intervals. Additionally, to select the best models, we used the highest AUC for FP-reduction classifiers, and the top global Dice for lesion segmentation models from CV. All these methods were integrated into a pipeline (*i.e*., LLSB_XPC, LLSB_LVC, and LLSB_CFPR) to fine-tune the thresholds *thr_e* and *thr_i* to maximize global Dice while preserving optimal lesion sensitivity from CV.

## Results

In this section, following RECIST 1.1 guidelines [3], we report the performance of our method using measurable lesions (≥ 10 mm in diameter). This approach prioritizes annotation reliability and ensures alignment with current clinical practices, where lesions smaller than 10 mm are typically excluded due to partial volume effects and high inter-radiologist variability. However, comprehensive results evaluating the pipeline on the full set of annotated lesions, including those < 10 mm, are provided in Supplementary Material, Section S3.

### Test performance

Table 2 provides the results of the proposed pipeline (LLSB_CFPR) and the different ablation versions of it on the test set of the DS-Fuse cohort. The LLSB_CFPR pipeline achieved the best performance, with a DSC of 75.6% (image level) and 81.0% (lesion level) for segmentation and an F1-score of 71.2% for lesion detection. Also, Table 2 compares these results with state-of-the-art networks trained on the same dataset as the LLSB pipeline. Among them, Swin-UNet achieved the highest lesion segmentation performance (70.6% DSC), while U-Net++ had the best lesion detection (63.7% F1-score).

**Table 2:** Results of the different lung lesions segmentation pipelines and alternative segmentation networks on the DS-Fuse test set

Method	Segmentation			Detection		
	DSC (image)	DSC (lesion)	Hd-95 (lesion)	F1	Precision	Recall
LLS (baseline)	0.692	0.800	8.560	0.609	0.482	0.827
	0.65–0.73	0.78–0.81	7.46–9.79	0.58–0.64	0.45–0.51	0.80–0.85
LLSB	0.746	0.810	7.440	0.680	0.567	0.849
	0.71–0.78	0.79–0.83	6.48–8.35	0.66–0.71	0.54–0.60	0.82–0.88
LLSB_XPC	0.755	0.810	7.440	0.702	0.599	0.849
	0.72–0.79	0.79–0.83	6.46–8.41	0.68–0.73	0.57–0.63	0.82–0.88
LLSB_LVC	0.748	0.810	7.440	0.690	0.585	0.847
	0.71–0.78	0.79–0.83	6.48–8.32	0.67–0.72	0.56–0.62	0.82–0.88
LLSB_CFPR	0.756	0.810	7.440	0.712	0.615	0.847
	0.72–0.79	0.79–0.83	6.49–8.47	0.68–0.73	0.58–0.64	0.82–0.87
UResNet	0.694	0.760	9.600	0.513	0.358	0.907
	0.66–0.73	0.74–0.78	8.31–10.9	0.49–0.54	0.34–0.38	0.89–0.93
U-Net++	0.607	0.750	9.840	0.638	0.518	0.830
	0.57–0.65	0.73–0.76	8.52–11.0	0.61–0.66	0.49–0.55	0.80–0.86
Swin-UNet	0.706	0.770	8.800	0.579	0.441	0.841
	0.67–0.74	0.75–0.78	7.70–10.0	0.55–0.60	0.42–0.47	0.81–0.87

Data are given as mean (upper row) and 95% confidence interval (lower row)

*CFPR* Cascade false-positive reduction, *Hd-95* 95%-percentile Hausdorff distance, *LLS* Lung lesion segmentation pipeline, *LLSB* Lung lesion segmentation pipeline with thoracic bounding box, *LVC* Lesion validator classifier, *XPC* Extrapulmonary classifier

**Fig. 5:**
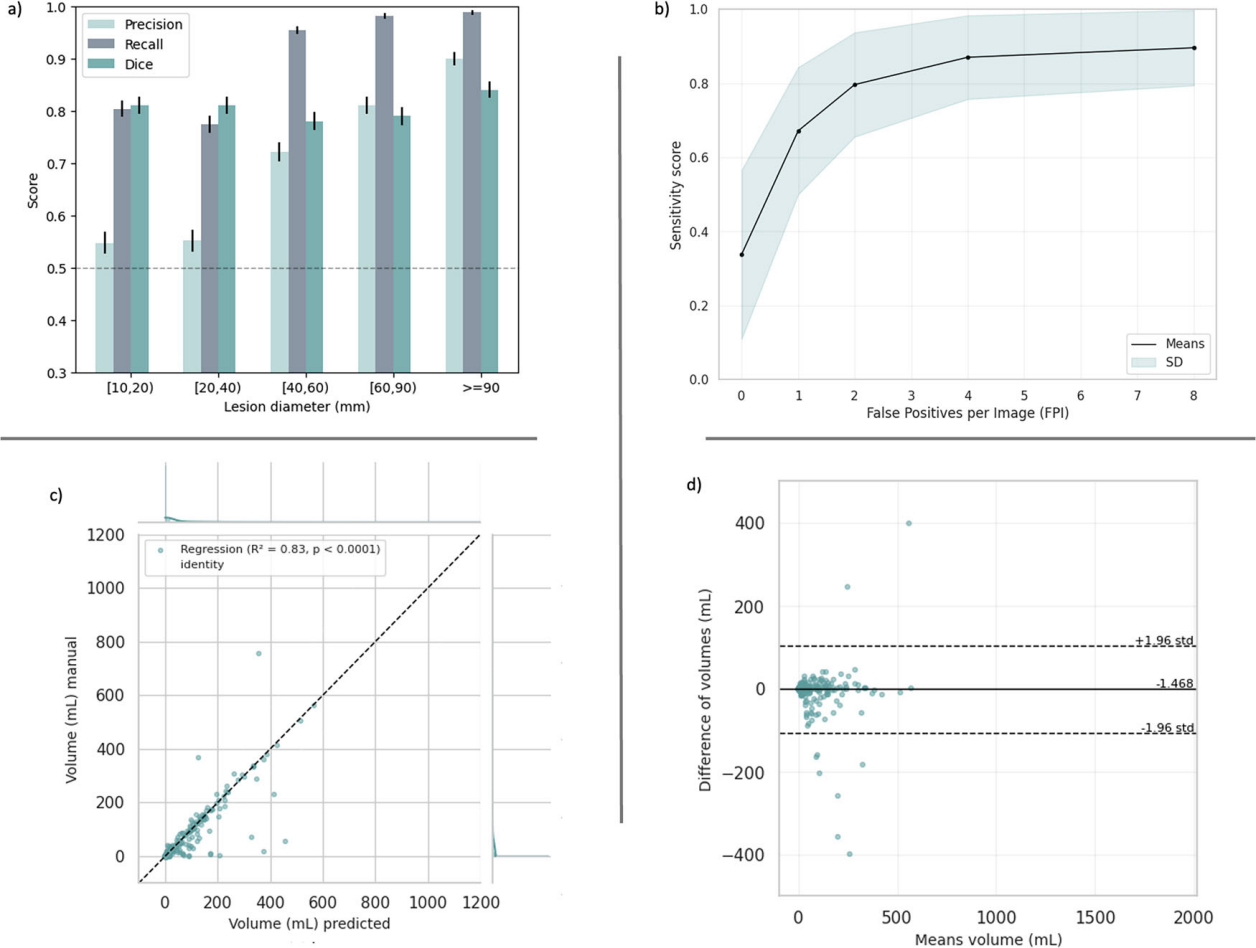
Test performance details of the LLSB_CFPR pipeline. At the top, we show performances by lesion size (**a**) and the free-response AUROC curve (**b**). **c**, **d** Illustrate lesion volumetry agreement between predicted and ground truth (manual)

Figure 5 provides further insights into the performance of the LLSB_CFPR pipeline. At the top, we illustrate the relationship between lesion detection and segmentation accuracy. The panel a shows consistently high sensitivity and DSC, typically exceeding 80%, across all detected lesion sizes. However, precision remains lower for lesions below 40 mm, highlighting further optimization. Panel b presents the sensitivity-to-FP ratio per CT scan, demonstrating a relevant average sensitivity of 80% with 2 FPs per patient. At the bottom of Fig. 5, we assess the agreement between predicted and manual lesion volume measurements. Panel C depicts a strong correlation between both measurements (*R*^*2*^ = 0.83, *p* < 0.0001). Panel d displays a Bland–Altman plot, highlighting a minimal bias (-0.001 L) and narrow limits of agreement (0.103, -0.106 L).

**Fig. 6:**
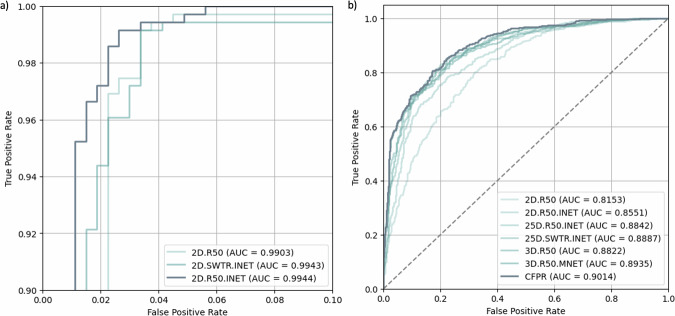
Comparative AUROC curve plots for XPC classifiers on the test partition of DS-FPX (**a**) and for all FP-reduction classifiers on the test DS-FP (**b**)

To assess the results of the proposed FP-reduction methods independently of the pipelines, Table 3 presents their performance on the DS-FP test cohort, which includes lesion candidates across the entire thoracic regions. The best-performing XPC classifier (2D.R50.INET), trained on the DS-FPX cohort to filter extrapulmonary lesion candidates, exhibited limited performance on DS-FP, yielding an AUROC of 71.1%. However, when tested on DS-FPX, XPC classifiers achieved AUROCs above 99%, demonstrating their effectiveness for their intended task (Fig. 6a). In contrast, LVC classifiers, trained on the DS-FP cohort and optimized to identify erroneous lesion segmentations within the lung parenchyma, demonstrated superior performance. The best LVC classifier (3D.R50.MNET) achieved an AUC of 89.4%. The proposed combined method (CFPR) outperformed all individual approaches (Fig. 6b), improving AUROC by 0.7%, and precision by 1.9%, while maintaining the same recall as the best LVC method.

**Table 3:** Results of the different FP reduction classifiers on the DS-FP test set cohort

Method	Classifiers	AUROC	Precision	Recall	Specificity
XPC	2D.R50	0.674	0.608	0.991	0.202
		0.64–0.70	0.57–0.63	0.98–0.99	0.17–0.23
	2D.R50.INET*	0.711	0.608	**0.995**	0.196
		0.68–0.74	0.57–0.63	0.98–1.0	0.16–0.23
	2D.SWTR.INET	0.691	0.609	0.992	0.204
		0.65–0.72	0.57–0.64	0.98–0.99	0.16–0.23
LVC	2D.R50	0.815	0.631	0.976	0.287
		0.79–0.84	0.60–0.66	0.96–0.98	0.24–0.32
	2D.R50.INET	0.855	0.728	0.902	0.578
		0.83–0.87	0.69–0.75	0.87–0.92	0.53–0.62
	25D.R50.INET	0.884	0.746	0.924	0.606
		0.86–0.90	0.71–0.77	0.90–0.94	0.56–0.64
	25D.SWTR.INET	0.889	0.73	0.948	0.560
		0.87–0.90	0.70–0.75	0.93–0.96	0.51–0.60
	3D.R50	0.882	0.705	0.943	0.505
		0.86–0.90	0.67–0.73	0.92–0.96	0.46–0.54
	3D.R50.MNET*	0.894	0.753	0.924	0.62
		0.87–0.91	0.72–0.78	0.90–0.94	0.57–0.66
CFPR	XPC|LVC	**0.901**	**0.772**	0.924	**0.659**
		0.88–0.91	0.74–0.80	0.90–0.94	0.61–0.70

Data are given as mean (upper row) and 95% confidence interval (lower row)

*CFPR* Cascade false-positive reduction, *INET* ImageNet, *LVC* Lesion validator classifier, *MNET* Medical Net, *R50* ResNet-50, *SWTR* Swin transformer, *XPC* Extrapulmonary classifier

* Classifiers marked with an asterisk achieved the best performance and were used for the CFPR

The bold values in the table indicates the highest scores in each column

### External validation performance

**Fig. 7:**
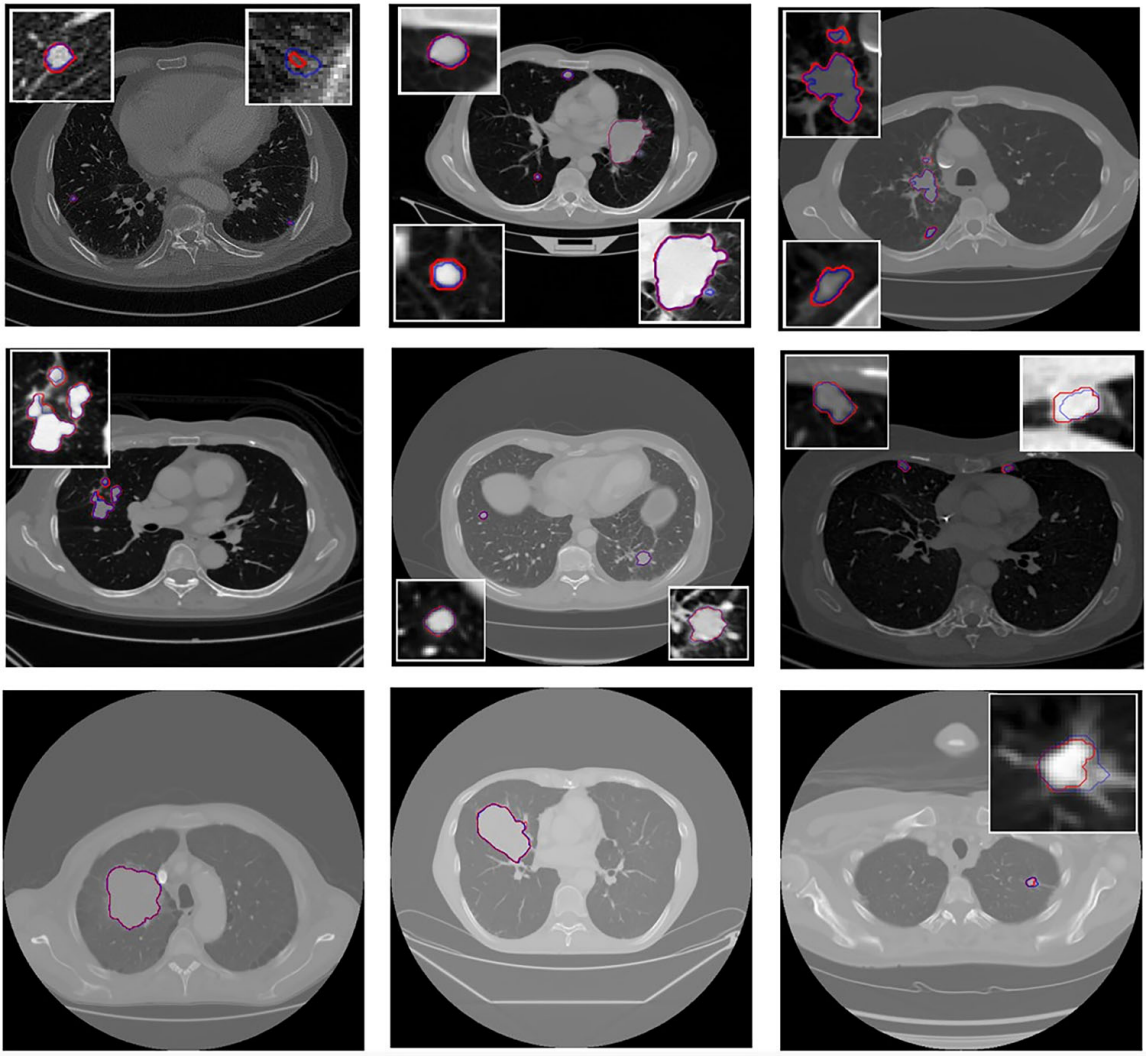
Cases from the external dataset showing predicted lesions (blue) by the LLSB-CFPR pipeline and ground truth lesions (red)

The LLSB_CFPR pipeline was externally validated on the DS-CHM dataset, achieving a DSC of 72.9% (95% confidence interval 69–77%) at the image level and 78.3% (76–81%) at the lesion level. For lesion detection, the pipeline obtained an F1-score of 73.3% (70–76%), with a precision of 64.5% (61–68%) and a recall of 84.9% (82–88%). Lesion volume quantification agreement with ground truth annotations reached a 0.59 of *R²* (*p* < 0.0001). Refer to Supplementary Material, Section S4 for further performance details. Figure 7 illustrates different qualitative test results of the proposed pipeline.

## Discussion

This study introduced LLSB-CFPR, a novel deep learning-based automated pipeline for lung cancer lesion segmentation in CT scans, designed to address key limitations of existing approaches [12–16, 18, 33]. First, it was developed and evaluated using a large and heterogeneous multi-center cohort comprising real-world clinical data from three distinct sources. This diversity enhances the model’s robustness and generalizability by capturing variations in imaging manufacturers, acquisition protocols, and lesion characteristics. Second, unlike conventional methods that focus solely on segmenting the primary lung cancer lesions, the proposed pipeline was designed to delineate all potential lung cancer lesions within a CT scan. This strategy enhances the clinical applicability of the tool by providing a more comprehensive representation of tumor burden, which is essential for disease monitoring, treatment planning, and longitudinal assessment.

The pipeline was evaluated on a test set comprising over 200 cases and on an independent external cohort consisting exclusively of real-world clinical cases. Notably, its performance was assessed on measurable lesions (≥ 10 mm in diameter) as defined by RECIST 1.1 [3], ensuring clinical relevance, avoiding ambiguity in ground-truth annotations, and alignment with standardized oncologic response criteria.

Particularly, the pipeline demonstrated a robust multi-lesion detection capability, achieving 85% sensitivity in both the test and external cohorts. In terms of segmentation performance, the pipeline attained an average Dice score of 76% and 73% across all cases in the test and external datasets, respectively. Notably, this performance exceeded that of a similar study [34]—which trained and evaluated a segmentation model using nnU-Net [17] on a large multi-center cohort—by 6% and 3% in the test dataset. Furthermore, for detected lesions, the pipeline consistently achieved a DSC near or above 80%, regardless of lesion sizes, across both validation cohorts, being especially significant for larger lesions, which pose a greater clinical burden for radiologists.

Regarding the balance between sensitivity and FPs, the pipeline reached an average sensitivity of 80% at 2 FPs per CT scan, outperforming a recent study [35] that reported 4 FPs per CT scan on a test set of 20 cases with multiple lung cancer lesions. Additionally, the same study reported a 34% volume agreement between predicted and physician-annotated lesions, whereas our method demonstrated an *R*^*2*^ exceeding 80% and 60% in both test and external cohorts. Bland–Altman analysis further confirmed the reliability of our approach, showing minimal bias and narrow agreement limits, reinforcing its potential for clinical implementation.

Further analysis of our results with existing studies remains challenging due to the scarcity of research addressing multi-lesion lung cancer segmentation. Therefore, we compared our approach with distinct state-of-the-art networks using the same dataset and comparable model configurations. Swin-UNet [32], the best-performing segmentation model, exhibited a 4% decrease in DSC, while UNet++ [31], which achieved the highest performance in lesion detection, showed a 4.2% drop in F1-score compared to the LLSB pipeline. Although these differences may appear modest in absolute terms, in the context of lung cancer lesion segmentation, even minor gains can be translated into clinically meaningful benefits, potentially improving the reliability of downstream tasks such as volumetric quantification, disease monitoring, and treatment planning [36].

Eventually, to gain deeper insights into the contribution of each component within the LLSB-CFPR pipeline, we conducted an ablation study. We began with a reference pipeline (LLS), that included only the lesion segmentation step. We then evaluated the LLSB pipeline, which introduced the thoracic bounding box step—a technique previously applied by Liu et al [33]. This addition led to a 5.4% increase in DSC (image-level) and a 7.1% improvement in the F1-score, underscoring the impact of this preprocessing step. Subsequently, we investigated the effect of integrating an FP-reduction step into the LLSB pipeline. This strategy has been commonly used in multi-instance lesion tasks, such as liver metastases segmentation [37], or lung nodule detection [38]. Traditional FP-reduction relies on a single classifier to filter lesion candidates within the target organ [19]. In our study, we proposed CFPR, an FP-reduction method composed of two distinct classifiers (XPC and LSV) that work in cascade, addressing different types of lesion candidates. We explored each of them individually within the LLSB_XPC and LLSB_LSV pipelines. The LLSB_XPC improved DSC by 0.9% and F1-score by 2.2% over LLSB, demonstrating that extrapulmonary FPs were a predominant error source and that XPC effectively filtered them while maintaining high recall rates. Conversely, the LLSB_LVC pipeline resulted in only a 0.2% increase in Dice and a 1% improvement in F1-score over LLSB, indicating the limited impact of LVC alone. Finally, the fully integrated LLSB_CFPR pipeline, using the CFPR approach, achieved the highest performance gains, with a 1% increase in DSC and a 3.2% improvement in the F1-score over LLSB. These results underscore the advantages of decomposing the problem into distinct tailored classifier types and cascading them to optimize overall FP reduction.

This study has several limitations. Although the proposed method demonstrates encouraging results for the underexplored task of multi-instance lung cancer segmentation for characterization, monitoring, and treatment follow-up, a comprehensive error analysis is essential prior to clinical deployment. This is crucial to ensure that segmentation tools achieve high precision and sensitivity in routine clinical practice. In this line, we observed specific anatomical regions susceptible to segmentation errors. FPs were frequently detected near the hilar vessels and along pleural surfaces, whereas false negatives tended to occur near the mediastinum. Similar error patterns in these anatomically complex regions were also reported in previous deep learning-based segmentation studies [35]. Supplementary Material Section S5 provides representative examples of these errors. Another limitation of this work is the reduced lesion-free CTs included in the training and test sets. Consequently, a systematic evaluation of the method’s performance in entirely lesion-free populations remains warranted. Furthermore, variability in lesion size, morphology, and location poses significant challenges for clinical application. Despite using a diverse cohort, consistent performance across all lesion subtypes, including rare and underrepresented cases, is essential. This requires rigorous external validation using large, heterogeneous, multi-institutional cohorts. While this study provides a solid foundation, extensive validation is still needed before clinical translation.

In conclusion, we presented an automatic pipeline for multi-instance lung cancer lesion segmentation, addressing an underexplored problem using multi-center, real-world data. The proposed method demonstrated robust performance when evaluated on both an independent test set and an external validation cohort, highlighting its potential to advance multi-lesion segmentation in heterogeneous lung cancer datasets.

## Supplementary information

**Additional file 1**: **Table S1.** Additional details of the external cohort (DS-CHM), including characteristics of CT scans and lesions. Values are presented as median (IQR range, 25%–75%). **Table S2.** Classifier setups for the XPC and LVC methods. The “Input” column denotes “I” for image and “M” for mask. “Chs” indicates the number of input channels. “Weights” specifies if the classifier was pretrained (ImageNet [19], MedicalNet [20]) or trained from scratch. Other columns are self-explanatory. **Table S3.** Evaluation results of the different pipelines using FP-reduction. Results are mean +/- stdev. **Table S4.** Test results of the different pipelines for multi-instance lung cancer lesion detection and segmentation. **Table S5.** Evaluation results of alternative segmentation networks. Results are in mean +/- stdev. **Table S6.** Test results of alternative segmentation networks. **Table S7.** Evaluation results of XPC classifiers in the DS-DFX training set. Results are in mean +/- stdev. **Table S8.** Evaluation results of LVC classifiers in the DS-FP training set. Results are in mean +/- stdev. **Figure S1.** External cohort performance details of the LLSB_CFPR pipeline. At the top we show performances stratified by lesion size (Left) and the FROC curve (Right). At the bottom, we illustrate lesion volumetry agreement between predicted and ground truth (manual). **Figure S2.** Example of two cases with missed lesions (FN). In the top image, the lesion is located in the hilar region; in the bottom image, the missed lesion is situated in the mediastinal region. **Figure S3.** Examples of segmentation failures on two CT scans. In the top image, two non-lesions were incorrectly segmented (FP) in the lung walls. In the bottom image, two true lesions were missed (FN) in the lung walls, while two others were correctly detected and segmented (TP).

## Data Availability

For data access, please contact the authors of the manuscript.

## Abbreviations

2D: Two-dimensional
3D: Three-dimensional
AUROC: Area under the receiver operating characteristics curve
CFPR: Cascade false positives reduction
CV: Cross-validation
DS: Data source
DSC: Dice similarity coefficient
FoV: Field of view
FP: False positive
Hd-95: 95th-percentile Hausdorff distance
INET: ImageNet
LLS: Lung lesion segmentation pipeline
LLSB: Lung lesion segmentation pipeline with thoracic bounding box
LVC: Lesion validator classifier
MNET: Medical Net
nnU-Net: No new U-Net
NSCLC: Non-small cell lung cancer
R50: ResNet-50
XPC: Extrapulmonary classifier
