# Supplementary material for: Advancing deep learning-based segmentation for multiple lung cancer lesions in real-world multicenter CT scans
Source: Eur Radiol Exp. 2025 Aug 18;9:78. doi: 10.1186/s41747-025-00617-7 (PMC12361585; doi:10.1186/s41747-025-00617-7)
Supplement: Supplementary file 1 — Additional file 1: Table S1. Additional details of the external cohort (DS-CHM), including characteristics of CT scans and lesions. Values are presented as median (IQR range, 25%–75%). Table S2. Classifier setups for the XPC and LVC methods. The “Input” column denotes “I” for image and “M” for mask. “Chs” indicates the number of input channels. “Weights” specifies if the classifier was pretrained (ImageNet [19], MedicalNet [20]) or trained from scratch. Other columns are self-explanatory. Table S3. Evaluation results of the different pipelines using FP-reduction. Results are mean +/- stdev. Table S4. Test results of the different pipelines for multi-instance lung cancer lesion detection and segmentation. Table S5. Evaluation results of alternative segmentation networks. Results are in mean +/- stdev. Table S6. Test results of alternative segmentation networks. Table S7. Evaluation results of XPC classifiers in the DS-DFX training set. Results are in mean +/- stdev. Table S8. Evaluation results of LVC classifiers in the DS-FP training set. Results are in mean +/- stdev. Figure S1. External cohort performance details of the LLSB_CFPR pipeline. At the top we show performances stratified by lesion size (Left) and the FROC curve (Right). At the bottom, we illustrate lesion volumetry agreement between predicted and ground truth (manual). Figure S2. Example of two cases with missed lesions (FN). In the top image, the lesion is located in the hilar region; in the bottom image, the missed lesion is situated in the mediastinal region. Figure S3. Examples of segmentation failures on two CT scans. In the top image, two non-lesions were incorrectly segmented (FP) in the lung walls. In the bottom image, two true lesions were missed (FN) in the lung walls, while two others were correctly detected and segmented (TP). [file 41747_2025_617_MOESM1_ESM.pdf]

Advancing deep learning-based segmentation for multiple lung cancer lesions in real-world multicenter CT scans

ELECTRONIC SUPPLEMENTARY MATERIAL

Eur Radiol Exp (2025) Rafael-Palou X, Jimenez-Pastor A, Marti-Bonmati L, Muñoz-Nuñez CF, Laudazi M, Alberich-Bayarri Á.

S1. External cohort details

The DS-CHM cohort, comprised of 188 cases of real-world data, was obtained from the ChAImeleon European project [22], with approval from the corresponding ethical committee. The data was collected between 2015 January and 2022 January; the age of the subjects was between 35 to 83 years with a female ratio of 60%.

Characteristic	CHM
**CT Scans**	
Total Count	188
**Lesions (All)**	
Total Count	623
Lesions per CT	1 *(1-3)*
Diameter (mm)	14 *(10-28.5)*
**Lesions (≥10 mm)**	
Total Count	496
Lesions per CT	1 *(1-2)*
Diameter (mm)	17.8 *(12.8-39.4)*
**Image Size (mm)**	
x-dimension	413 *(375-454)*
y-dimension	413 *(375-454)*
z-dimension	616 *(327-624)*
**Voxel Size (mm)**	
x-dimension	0.73 *(0.59-0.80)*
y-dimension	0.73 *(0.59-0.80)*
z-dimension	1.5 *(0.5-1.5)*

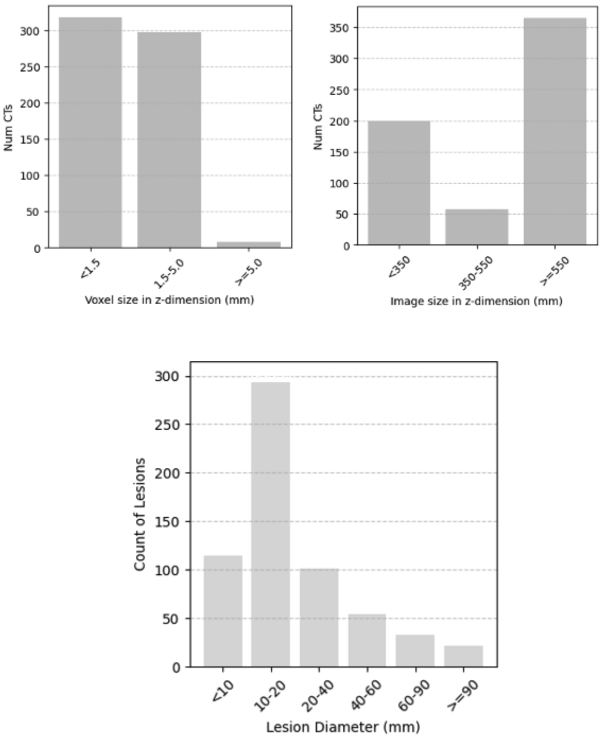

Table S1. Additional details of the external cohort (DS-CHM), including characteristics of CT scans and lesions. Values are presented as median (IQR range, 25%-75%).

S2. Experiments setup

2.1 Alternative segmentation networks

Following, we describe the training setup of the alternative networks (UResNet [27], U-Net++ [28] and Swin-UNet [29]) used for lung cancer lesion segmentation. Those settings were designed to be as much consistent as the ones used to build the segmentation model of our pipeline.

- **Input patches**: Randomly extracted patches of size 128x128x128. Smaller crops were padded to match the target dimensions.
- **Intensity normalization**: All patches were clipped to the Hounsfield unit (HU) range of [-100, 500] and normalized to [0,1].
- **Data augmentation**: Applied with a probability of 15%, including 3D rotation (+/- 65 degrees), zoom transformations (+/- 0.1), Gaussian noise and flipping across all spatial axes.

The networks were extracted and trained using the MONAI library* (version 1.2.0), with default configurations for parameters not explicitly specified. The details of each architecture are as follows:

- **UResNet** [27]: Composed of 5 layers, each performing down/up sampling by a factor of 2, with 2-convolution residual units per layer. The encoder feature sizes were [16, 32, 64, 128 and 256]. Instance-based normalization and dropout (20% of probability) were used.
- **U-Net++** [28]: Consisted of layers with encoder feature sizes [32, 32, 64, 128 and 256]. Batch normalization and dropout (20%) were applied. No deep supervision was employed.
- **Swin-UNet** [29]: Configured with an initial feature size of 48 and two layers per stage across four stages. The number of self- attention heads per stage was [3, 6, 12 and 24]. No dropout was applied.

All networks were trained from scratch using the Adam optimizer for 500 epochs, with an initial learning rate of 1e-4. The loss function was a weighted combination of Dice loss and cross-entropy loss, both given equal importance.

(*) Cardoso MJ, Li, W, Brown R, Ma N, Kerfoot E, Wang Y, et al (2022). MONAI: An open-source framework for deep learning in healthcare. https://doi.org/https://doi.org/10.48550/arXiv.2211.02701

2.2 FP-reduction networks

XPC Classifiers

Method	Network	Filter	Size	Chs	Plane	Input	Weights
2D.R50	ResNet-50	2D	128	3	Coronal	[I,I,M]	Scratch
2D.R50.INET	ResNet-50	2D	128	3	Coronal	[I,I,M]	ImageNet
2D.SWTR.INET	Swin Transformer	2D	128	3	Coronal	[I,I,M]	ImageNet

LVC Classifiers

Method	Network	Filter	Size	Chs	Plane	Input	Weights
2D.R50	ResNet-50	2D	128	3	Transversal	[I,I,M]	Scratch
2D.R50.INET	ResNet-50	2D	128	3	Transversal	[I,I,M]	ImageNet
2'5D.R50.INET	ResNet-50	2D	128	6	Transversal (t) Coronal (c) Sagittal (s)	[I_t,I_c,I_s, M_t,M_c,M_s]	ImageNet
2'5D.SWTR.INET	Swin Transformer	2D	128	6	Transversal (t) Coronal (c) Sagittal (s)	[I_t,I_c,I_s, M_t,M_c,M_s]	ImageNet
3D.R50	ResNet-50	3D	96	2	Full	[I,M]	Scratch
3D.R50.MNET	ResNet-50	3D	96	2	Full	[I,M]	MedicalNet

Table S2. Classifier setups for the XPC and LVC methods. The "Input" column denotes "I" for image and "M" for mask. "Chs" indicates the number of input channels. "Weights" specifies if the classifier was pretrained (ImageNet [18], MedicalNet [19]) or trained from scratch. Other columns are self-explanatory.

S3. Complete cohort results

In this section we provide the evaluation and test results of the proposed method for multi-instance lung cancer lesion detection and segmentation. These results were obtained using **all annotated lesions** (including <10 mm in diameter) from the DS-Fuse cohort.

3.1 Pipelines performance

Table-S3 presents the evaluation and fine-tuning results of different pipelines (LLS, LLSB, LLSB_XPC, LLSB _LVC and LLSB _CFPR) obtained from a 5-fold CV on the DS-Fuse training data. Pipelines incorporating an FP-reduction method (i.e., XPC, LVC or CFPR) were evaluated across various classification thresholds to identify the optimal ones.

			Evaluation			
			Dice	Detection		
Pipeline	thr_p	thr_i	(CT)	F1	Prec	Rec
**LLS**	**-**	**-**	0.627±0.027	0.480±0.044	0.349±0.048	0.778±0.018
**LLSB**	**-**	**-**	0.690±0.007	0.576±0.037	0.465±0.052	0.765±0.014
**LLSB_XPC**	**0.01**	**-**	0.704±0.006	0.608±0.033	0.507±0.049	**0.764±0.010**
LLSB_XPC	0.05	-	0.706±0.006	0.612±0.033	0.513±0.050	0.763±0.014
LLSB_XPC	0.10	-	0.708±0.006	0.614±0.033	0.515±0.049	0.763±0.014
LLSB_XPC	0.50	-	0.708±0.005	0.616±0.033	0.518±0.050	0.762±0.014
LLSB_LVC	-	0.10	0.691±0.007	0.589±0.037	0.481±0.053	0.763±0.015
**LLSB_LVC**	**-**	**0.20**	0.693±0.007	0.611±0.037	0.513±0.057	**0.763±0.015**
LLSB_LVC	-	0.30	0.695±0.007	0.625±0.036	0.535±0.059	0.757±0.013
LLSB_LVC	-	0.50	0.699±0.010	0.651±0.033	0.580±0.060	0.745±0.013
LLSB_CFPR	0.01	0.10	0.705+/-0.006	0.618+/-0.034	0.521+/-0.052	0.763+/-0.015
**LLSB_CFPR**	**0.01**	**0.20**	0.706+/-0.005	0.638+/-0.034	0.551+/-0.056	**0.763+/-0.015**
LLSB_CFPR	0.01	0.30	0.707+/-0.006	0.650+/-0.035	0.572+/-0.058	0.756+/-0.013
LLSB_CFPR	0.01	0.50	0.707+/-0.008	0.673+/-0.034	0.617+/-0.062	0.745+/-0.013
LLSB_CFPR	0.05	0.10	0.706+/-0.006	0.621+/-0.034	0.526+/-0.052	0.763+/-0.014
LLSB_CFPR	0.05	0.20	0.708+/-0.006	0.641+/-0.034	0.557+/-0.056	0.761+/-0.015
LLSB_CFPR	0.05	0.30	0.708+/-0.006	0.653+/-0.035	0.577+/-0.059	0.756+/-0.013
LLSB_CFPR	0.05	0.50	0.709+/-0.008	0.676+/-0.034	0.622+/-0.063	0.744+/-0.013
LLSB_CFPR	0.10	0.10	0.708+/-0.006	0.623+/-0.034	0.528+/-0.052	0.763+/-0.014
LLSB_CFPR	0.10	0.20	0.709+/-0.006	0.643+/-0.034	0.559+/-0.055	0.761+/-0.015
LLSB_CFPR	0.10	0.30	0.710+/-0.006	0.654+/-0.034	0.579+/-0.058	0.756+/-0.013
LLSB_CFPR	0.10	0.50	0.711+/-0.008	0.677+/-0.033	0.624+/-0.062	0.744+/-0.013
LLSB_CFPR	0.50	0.10	0.708+/-0.005	0.624+/-0.034	0.531+/-0.052	0.761+/-0.015
LLSB_CFPR	0.50	0.20	0.710+/-0.004	0.644+/-0.034	0.561+/-0.056	0.759+/-0.015
LLSB_CFPR	0.50	0.30	0.710+/-0.005	0.655+/-0.034	0.582+/-0.059	0.754+/-0.013
LLSB_CFPR	0.50	0.50	0.711+/-0.007	0.678+/-0.033	0.627+/-0.063	0.742+/-0.013

Table S3. Evaluation results of the different pipelines using FP-reduction. Results are mean +/- stdev.

As expected, the LLS pipeline demonstrated the poorest segmentation performance (Dice coefficient at the CT level) and the lowest detection metric (F1-score), despite having the highest recall (77.8%). The other pipelines reported slightly lower recall- decreasing by 1.3 to 1.5% compared to LLS- but achieved significantly higher Dice and F1 scores. Notably, our proposed method LLSB_CFPR, outperformed all others, achieving the highest Dice score (70.6%) and F1-score (63.8%).

Test performance

Following we show the results of the different pipelines on the test partition of the DS-Fuse cohort. The best pipeline was LLSB_CFPR achieving a 74% Dice coefficient (CT level) segmentation performance and a 64.6% of F1-score with 83.9% of sensitivity score for detecting multi-instance lung cancer lesions.

Pipeline	thr_e	thr_i	Dice (CT)	Detection F1	Detection Prec	Detection Rec
LLS	-	-	0.669	0.523	0.381	0.830
LLSB	-	-	0.728	0.589	0.452	**0.844**
LLSB_XPC	0.01	-	0.737	0.612	0.479	**0.844**
LLSB_LVC	-	0.2	0.732	0.626	0.499	0.839
**LLSB_CFPR**	0.01	0.2	**0.740**	**0.646**	**0.525**	0.839

Table S4. Test results of the different pipelines for multi-instance lung cancer lesion detection and segmentation.

3.2 Alternative segmentation networks

Table-S5 shows the evaluation results of the alternative lung cancer segmentation networks obtained from CV on the DS-FP training data.

Pipeline	Dice (CT)	Detection F1	Detection Prec	Detection Rec
UResNet	0.648 ± 0.022	0.37 ± 0.009	0.237 ± 0.007	**0.840 ± 0.012**
U-Net++	0.554 ± 0.022	**0.533 ± 0.011**	**0.397 ± 0.011**	0.810 ± 0.013
Swin_UNet	**0.679 ± 0.022**	0.468 ± 0.011	0.329 ± 0.010	0.809 ± 0.013

Table S5. Evaluation results of alternative segmentation networks. Results are in mean +/- stdev.

In Table-S6 we present the performance of these methods on the test set partition of DS-Fuse.

Method	Dice (CT)	Detection F1	Detection Prec	Detection Rec
UResNet	0.668	0.342	0.212	**0.881**
U-Net++	0.579	**0.479**	**0.337**	0.823
Swin_UNet	**0.684**	0.379	0.244	0.845

Table S6. Test results of alternative segmentation networks

3.3 FP-reduction

Next table shows the evaluation results of the XPC classifiers obtained from a 5-fold CV on the DS-FPX training data.

		Evaluation			
Method	Classifiers	F1	AUC	Precision	Recall
XPC	2D.R50	0.968±1.140	0.995±0.277	0.957±1.914	0.980±1.195
	**2D.R50.INET***	**0.987**±**0.421**	**0.998**±**0.133**	0.980±0.820	**0.992**±**0.402**
	2D.SWTR.INET	0.986±0.573	0.998±0.180	**0.983**±**0.915**	0.991±0.802

Table S7. Evaluation results of XPC classifiers in DS-DFX training set. Results are in mean +/- stdev.

Table-S8 details the evaluation results of the LVC classifiers obtained from a 5-fold CV on the DS-FP training data.

		Evaluation			
Method	Classifiers	F1	AUC	Precision	Recall
LVC	2D.R50	0.678±4.751	0.778±2.881	0.621±8.143	0.759±5.082
	2D.R50.INET	0.679±2.935	0.785±2.109	0.711±6.149	0.658±6.439
	25D.R50.INET	0.754±2.174	0.776±2.094	0.706±5.146	0.814±3.719
	25D.SWTR.INET	0.765±3.126	0.786±2.750	0.712±5.168	0.831±2.272
	3D.R50	0.765±3.291	0.783±3.273	0.701±5.105	**0.844**±**3.355**
	**3D.R50.MNET***	**0.775**±**3.623**	**0.797**±**2.892**	**0.732**±**4.148**	0.823±3.186

Table S8. Evaluation results of LVC classifiers in DS-FP training set. Results are in mean +/- stdev.

S4. External validation results

Following Figure-S1 provides further details regarding the performance of the LLSB_CFPR pipeline on the external cohort (DS_CHM). Results are only considering measurable lesions.

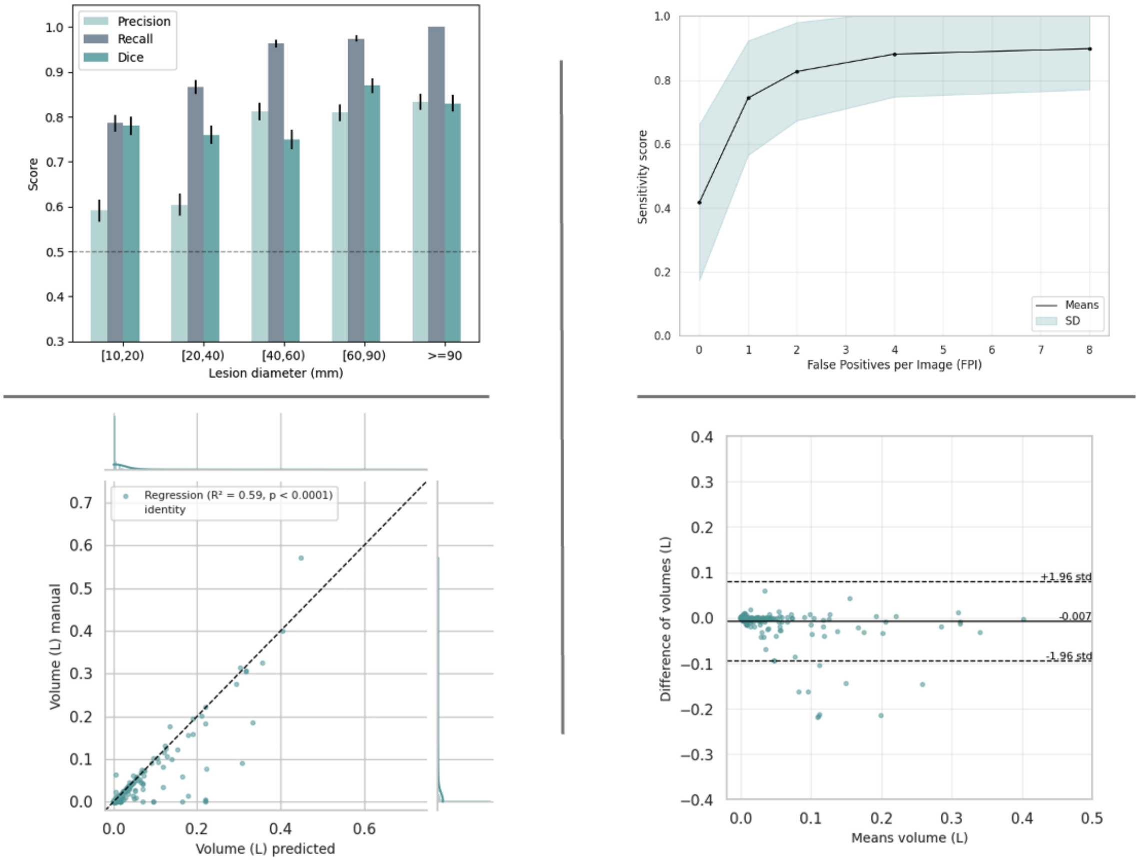

Figure S1. External cohort performance details of the LLSB_CFPR pipeline. At the top we show performances stratified by lesion size (Left) and the FROC curve (Right). At the bottom, we illustrate lesion volumetry agreement between predicted and ground truth (manual).

S5. Representative segmentation failures

Following images provide examples of areas or regions with representative segmentation failures. On the left we show the axial slice overlayed with the predicted lesion segmentation mask (yellow). On the right the original CT slice overlayed with the annotated lesion (red) surrounded by a yellow bounding box.

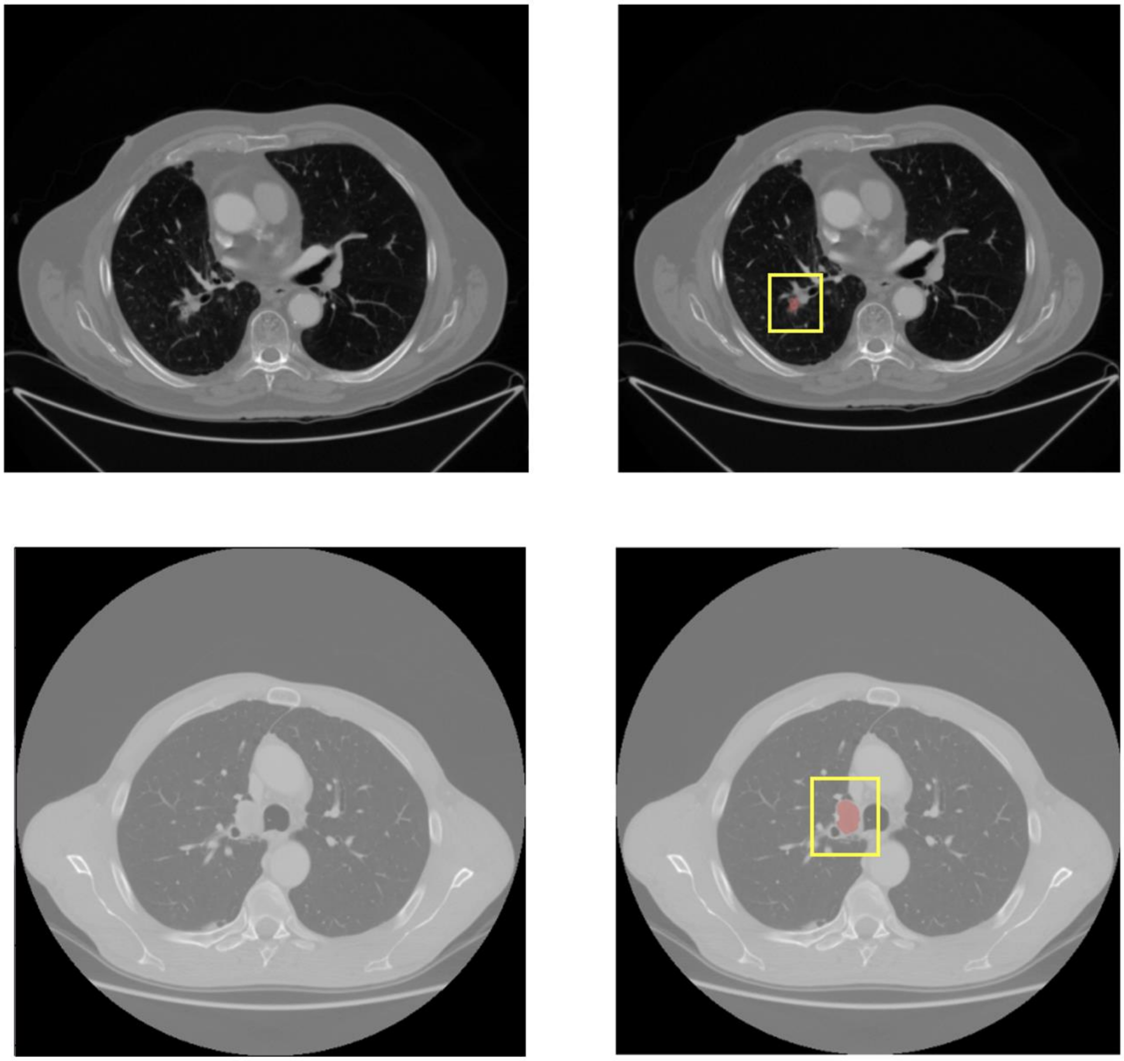

Figure S2. Example of two cases with missed lesions (FN). In the top image, the lesion is located in the hilar region; in the bottom image, the missed lesion is situated in the mediastinal region.

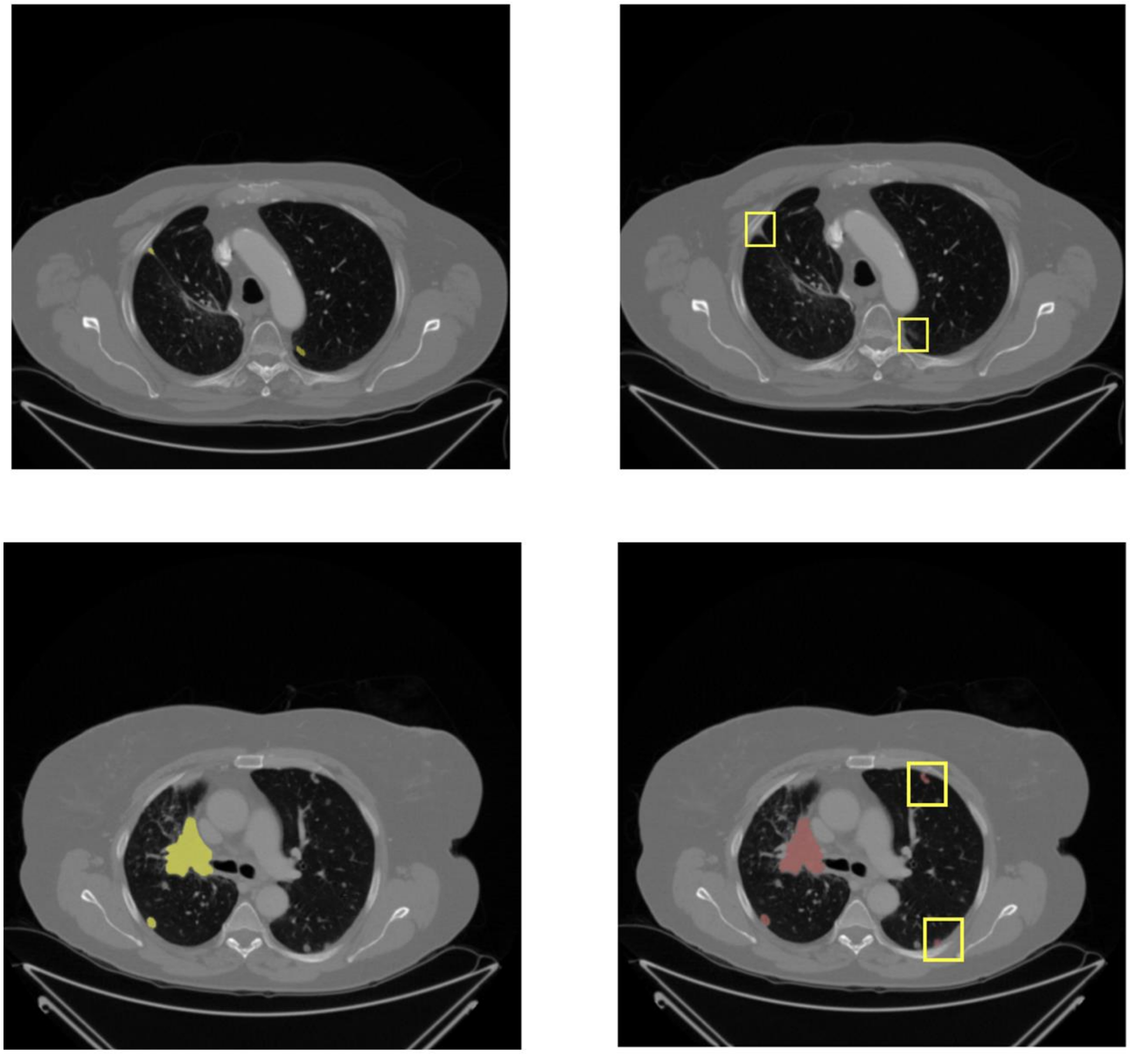

Figure S3. Examples of segmentation failures on two CT scans. In the top image, two non-lesions were incorrectly segmented (FP) in the lung walls. In the bottom image, two true lesions were missed (FN) in the lung walls, while two others were correctly detected and segmented (TP).

S6. CLAIM checklist

Section / Topic	No.	Item	Page / Line	No	NA
TITLE / ABSTRACT					
	1	Identification as a study of AI methodology, specifying the category of technology used (e.g., deep learning)	**p.1 lines 1-2**		
ABSTRACT					
	2	Summary of study design, methods, results, and conclusions	**p.1 lines 15-30**		
INTRODUCTIO N					
	3	Scientific and/or clinical background, including the intended use and role of the AI approach	**p.4 lines 89-109**		
	4	Study aims, objectives, and hypotheses	**p.4 lines 110-119**		
METHODS					
Study Design	5	Prospective or retrospective study	**p.4 line 122**		
	6	Study goal	**p.4 lines 122-124**		
Data	7	Data sources	**p.5 lines 127-128**		
	8	Inclusion and exclusion criteria	**p.5 lines 128-137**		
	9	Data pre-processing	**p.5 138-147**		

	**10**	Selection of data subsets	**p.5** **lines 128-137**		
	**11**	De-identification methods			**x**
	**12**	How missing data were handled			**x**
	**13**	Image acquisition protocol	**p.5** **lines 150-151**		
Reference Standard	**14**	Definition of method(s) used to obtain reference standard	**p.5** **lines 142-143**		
	**15**	Rationale for choosing the reference standard	**p.5** **lines 142-145**		
	**16**	Source of reference standard annotations	**p.5** **lines 142-143**		
	**17**	Annotation of test set	**p.5** **lines 155-160**		
	**18**	Measures of inter- and intra-rater variability of features described by the annotators	**p.5** **lines 143-147**		
Data Partitions	**19**	How data were assigned to partitions	**p.5** **lines 148-152**		
	**20**	Level at which partitions are disjoint	**p.5** **lines 148-152**		
Testing Data	**21**	Intended sample size	**p.5** **lines 155-160**		

Section / Topic	No.	Item	Page / Line	No	NA
Model	**22**	Detailed description of model	**p.6-7 Lines 162-215**		
	**23**	Software libraries, frameworks, and packages	**p.6 lines 170,181**		
	**24**	Initialization of model parameters	**p.7 lines 212-215**		
Training	**25**	Details of training approach	**p.7-8 Lines 217-253**		
	**26**	Method of selecting the final model	**p.8-9 lines 270-2573**		
	**27**	Ensembling techniques			**x**
Evaluation	**28**	Metrics of model performance	**p.8-9 lines 256-273**		
	**29**	Statistical measures of significance and uncertainty	**p.8-9 lines 269-270**		
	**30**	Robustness or sensitivity analysis		**x**	
	**31**	Methods for explainability or interpretability		**x**	
	**32**	Evaluation on internal data	**p.8-9 Lines 256-273**		
	**33**	Testing on external data	**p.5**		

			**Lines 155-160**		
	**34**	Clinical trial registration	**p.3**<br>**lines 64-67**		
**RESULTS**					
Data	**35**	Numbers of patients or examinations included and excluded	**p.9**<br>**lines 276-281**		
	**36**	Demographic and clinical characteristics of cases in each partition	**p.5**<br>**Lines 128-137**		
Model performance	**37**	Performance metrics and measures of statistical uncertainty	**p.9-10**<br>**282-314**		
	**38**	Estimates of diagnostic performance and their precision			**x**
	**39**	Failure analysis of incorrect results	**p.11**<br>**376-378**		
**DISCUSSION**					
	**40**	Study limitations	**p.11-12**<br>**Lines 372-384**		
	**41**	Implications for practice, including intended use and/or clinical role	**p.11-12**<br>**lines 371-384**		
**OTHER INFORMATION**					
	**42**	Provide a reference to the full study protocol or to additional technical details	**p.3**<br>**lines 64-67**		

	**43**	Statement about the availability of software, trained model, and/or data	**p.3 Lines 72**		
	**44**	Sources of funding and other support; role of funders	**p.3 Lines 77**		

* Indicate page and/or line number for each checklist item that is present. NA = not applicable.
